# Multidisciplinary approach is associated with improved survival of hepatocellular carcinoma patients

**DOI:** 10.1371/journal.pone.0210730

**Published:** 2019-01-14

**Authors:** Dong Hyun Sinn, Gyu-Seong Choi, Hee Chul Park, Jong Man Kim, Honsoul Kim, Kyoung Doo Song, Tae Wook Kang, Min Woo Lee, Hyunchul Rhim, Dongho Hyun, Sung Ki Cho, Sung Wook Shin, Woo Kyoung Jeong, Seong Hyun Kim, Jeong Il Yu, Sang Yun Ha, Su Jin Lee, Ho Yeong Lim, Kyunga Kim, Joong Hyun Ahn, Wonseok Kang, Geum-Youn Gwak, Yong-Han Paik, Moon Seok Choi, Joon Hyeok Lee, Kwang Cheol Koh, Jae-Won Joh, Hyo Keun Lim, Seung Woon Paik

**Affiliations:** 1 Department of Medicine, Samsung Medical Center, Sungkyunkwan University School of Medicine, Seoul, Korea; 2 Department of Surgery, Samsung Medical Center, Sungkyunkwan University School of Medicine, Seoul, Korea; 3 Department of Radiation Oncology, Samsung Medical Center, Sungkyunkwan University School of Medicine, Seoul, Korea; 4 Department of Radiology and Center for Imaging Science, Samsung Medical Center, Sungkyunkwan University School of Medicine, Seoul, Korea; 5 Department of Pathology, Samsung Medical Center, Sungkyunkwan University School of Medicine, Seoul, Korea; 6 Statistics and Data Center, Research Institute for Future Medicine, Samsung Medical Center, Seoul, Korea; 7 Department of Health Sciences and Technology, SAIHST, Sungkyunkwan University, Seoul, Korea; Yonsei University College of Medicine, REPUBLIC OF KOREA

## Abstract

**Background:**

Given the complexity of managing hepatocellular carcinoma (HCC), a multidisciplinary approach (MDT) is recommended to optimize management of HCC patients. However, evidence suggesting that MDT improves patient outcome is limited.

**Methods:**

We performed a retrospective cohort study of all patients newly-diagnosed with HCC between 2005 and 2013 (n = 6,619). The overall survival (OS) rates between the patients who were and were not managed via MDT were compared in the entire cohort (n = 6,619), and in the exactly matched cohort (n = 1,396).

**Results:**

In the entire cohort, the 5-year survival rate was significantly higher in the patients who were managed via MDT compared to that of the patients who were not (71.2% vs. 49.4%, P < 0.001), with an adjusted hazard ratio (HR) of 0.47 (95% confidence interval [CI]; 0.41–0.53). In the exactly matched cohort, the 5-year survival rate was higher in patients who were managed via MDT (71.4% vs. 58.7%, P < 0.001; HR [95% CI] = 0.67 [0.56–0.80]). The survival benefit of MDT management was observed in most pre-defined subgroups, and was especially significant in patients with poor liver function (ALBI grade 2 or 3), intermediate or advanced tumor stage (BCLC stage B or C), or high alphafetoprotein levels (≥200 ng/ml).

**Conclusion:**

MDT management was associated with improved overall survival in HCC patients, indicating that MDT management can be a valuable option to improve outcome of HCC patients. This warrants prospective evaluations.

## Introduction

Hepatocellular carcinoma (HCC) is the third most common cause of cancer death worldwide with increasing mortality rate in many countries [1–3]. HCC usually develops in patients with liver disease that compromises liver function [4]. Many HCC patients suffer from decreased liver function, and sometimes liver failure is the cause of mortality without cancer progression [5]. Hence, the prognosis of patients with HCC is complex by the interplay of tumor burden and liver function [6,7]. HCC management is complex, as one should carefully assess not only the risks and benefits of treatment on tumor, but also its effect on liver function. HCC is also notorious for its high recurrence rate even after curative treatment for early-stage tumor [8]. Hence, while managing patients with HCC, one should consider possibility of recurrence and available therapeutic option at the time of recurrence. Liver transplantation (LT) is a highly effective treatment option for HCC [9], and can dramatically change the prognosis of patients. However, donor availability differs by patient and regions, making things more complex. Recent advances in HCC treatment provided multiple potentially efficacious treatments, but, randomized controlled trials comparing between treatments are largely limited [7]. Several HCC guidelines have been published to facilitate the selection of optimal management [6,7,10], however, these guidelines have both similarities and dissimilarities due to the geographic differences, available resources, and lack of high level of evidences [11], and are not followed well in real-life clinical practice [12].

Multidisciplinary approach is a strategy that can help cope with escalating complexity in health care [13]. Multidisciplinary tumor board (MDT) approach are defined as an alliance of all medical and health care professionals related to a specific tumor disease whose approach to cancer care is guided by their willingness to agree on evidence-based clinical decisions and to co-ordinate the delivery of care at all stages of the process, encouraging patients in turn to take an active role in their care [14]. MDT management has been suggested as a tool to resolve a complex situation, where unpredictability, paradox and unknowable things remain [13,15]. HCC management is very complex, and hence, management via MDT may be a valuable option to provide optimal level of patients care [7,16]. However, MDT with participation by experts from multiple academic disciplines requires additional medical resources and time per patient, which leads to significant healthcare burden and high cost [17]. Several previous studies suggested that MDT management for HCC may improve survival [18–24], yet is limited by analyzing small number of patients [18–22], comparing patients who were treated in different time period [18,19,22], including those only received MDT management [20,21,23,24], or including those who received curative treatment only [20]. Recently, Serper et al., reported that MDT management was associated with reduced mortality, by analyzing 3,988 patients who received care through the Veterans Administration in involving 128 centers in U.S. [25]. However, survival advantage from MDT management might have been from receiving HCC care in high-volume center, where MDT management was available. Therefore, whether MDT management is necessary despite requiring more medical resources remains to be determined. In this study, we assessed whether management via MDT leads to improved survival in HCC patients.

## Materials and methods

### Study design and setting

This is a retrospective cohort study based on prospective HCC registry that has recorded clinical characteristics, tumor characteristics, and treatment information of newly diagnosed HCC patients since 2005 at Samsung Medical Center, Seoul, South Korea. A total of 6,619 consecutive patients with treatment naïve, newly diagnosed HCC were registered at Samsung Medical Center HCC registry between January 2005 and December 2013, and included in this study. During the study period, a total of 738 patients (11.1%, 738/6,619 patients) had MDT management (median: 1.4 times, min-max: 1–4 per patient). The Institutional Review Board of the Samsung Medical Center approved this study and waived the requirement for informed consent as we used only de-identified data routinely collected during hospital visits.

### MDT meeting

MDT for HCC patients was started at March 2005 at our institution. MDT meeting was held once a week. The MDT member comprised of hepatologists, surgeons, diagnostic radiologists, interventional radiologists specialized at local ablation therapies, interventional radiologists specialized at transarterial embolotherapies, radiation oncologists, medical oncologists, pathologists and coordinators. The decision to present a patient case for discussion at MDT was at the discretion of the physician in charge of the patient, usually by hepatologist or surgeons. The meeting was scheduled for an hour. As there was time limitation, the maximum number to be discussed at the MDT was 15 cases per each meeting, on a first comes first served basis. There was no specific requirement for a case to be discussed at the MDT meeting. However, usually difficult cases (e.g., tumor located in segment 1, hilar area, or near major vessels) are discussed at the MDT meeting.

At the MDT meeting, the physician in charge of the patient presented reason why cases were brought to the MDT meeting for a discussion. It was followed by image review by diagnostic radiologists, and then open discussion was performed. Even after discussion, there were some cases with controversies. Some cases were re-scheduled for re-discussion after further work-up procedures. Some cases in whom multiple treatment options were available and agreement on the best option was not made, the final decision was left to the physician in charge of the patient.

### Endpoints and variables

The primary outcome was overall survival (OS). Survival time was defined from the date of HCC diagnosis until the date of death or last hospital visit (assessed at March 15, 2017) whichever comes first. Exposure was the MDT management. For the confounders, the following variables were used: age at diagnosis, sex, year of diagnosis, etiology, Child-Pugh class, the Barcelona Clinic Liver Cancer (BCLC) stage, alpha-fetoprotein (AFP), the protein induced by vitamin K absence or antagonist-II (PIVKA-II), and initial treatment modality. In addition, we calculated albumin-bilirubin (ALBI) score using the following formula, −0.085 × (albumin g/l) + 0.66 × log (bilirubin μmol/l) [26]. Based on ALBI score, we classified the patients into three groups according to previously defined cut-offs: ALBI grade 1 (≤ −2.60), grade 2 (> −2.60 to −1.39) and grade 3 (> −1.39) [26]. We also classified patients whether patients received BCLC guideline recommended therapy, defined as receiving resection/ablation/LT for BCLC stage 0 or A, TACE for BCLC stage B, sorafenib for BCLC stage C and best supportive care for BCLC stage D.

### Exactly matched cohort

To balance the characteristics between the patients who were and were not managed via MDT, we generated the exactly matched cohort. For the exactly matched cohort, the following variables were categorized. Age was categorized into four categories; <50, 50–59, 60–69 and ≥70 years. The year of diagnosis was categorized into three groups; 2005–2007, 2008–2010 and 2011–2013. Etiology was categorized into two groups; hepatitis B-related HCC and others. For liver function estimate, we used ALBI grade as a matching variable. Serum AFP level was categorized into two groups at a cutoff point of 200 ng/mL. The cutoff point of AFP was selected after the Receiver Operating Curve (ROC) analysis at the point that can maximize survival difference between the two groups (low AFP vs. high AFP). Then, age groups (in four category), sex (men vs. women), year of diagnosis (2005–2007, 2008–2010, and 2011–2013), etiology (hepatitis B virus vs. other), ALBI grade (grade 1, 2, and 3), BCLC stage (stage 0, A, B, C, and D), AFP levels (<200 and ≥ 200 mg/dl), and LT during follow-up were exactly matched between patients with MDT management and patients without MDT management in a 1:1 ratio. The exactly matched cohort was comprised of 698 HCC patients with MDT management and 698 HCC patients without MDT management.

### Statistical analysis

We compared the variables between patients who were and were not managed by MDT in overall cohort. Difference in treatment choice between MDT and non-MDT group were also compared. Difference in OS was calculated using the Kaplan-Meier method and compared using the log-rank test. Hazard ratio (HR) for mortality by MDT management was assessed in un-adjusted and multivariable-adjusted model using Cox-proportional hazard model. Then, we compared OS between the patients who were and were not managed by MDT using exact matching cohort. Additionally, we performed stratified analyses to evaluate the differences in the OS via MDT in pre-specified subgroups, defined by age (<60, and ≥60 years), sex, year of diagnosis (2005–2007, 2008–2010, and 2011–2013), etiology (hepatitis B, and others), ALBI grade (grade 1, 2, and 3), BCLC stage (0, A, B and C), and AFP levels (<200, and ≥200 ng/mL). P-values of less than 0.05 were considered statistically significant.

## Results

The baseline characteristics of 6,619 HCC patients (median age: 57.0 years, men: 5,287 [79.9%], hepatitis B: 5,029 [76.0%]) are summarized according to the MDT management in Table 1. Patients who had MDT management were older, and included a higher number of men, had more preserved liver function, less advanced tumor stage, lower levels of AFP and PIVKA-II levels. Proportions of the patients who underwent resection, ablation or LT as a first-line treatment were similar for those who were managed via MDT than those who did not (49.2% vs.50.3%), but more patients received transarterial chemoembolization or other treatments (50.7% vs. 43.2%), and less patients received the best supportive care (0.1% vs. 6.4%) for those who were managed via MDT. There was no difference in terms of etiology and LT during follow-up.

**Table 1:** Baseline characteristics.

	With MDT care (n = 738)	Without MDT care (n = 5,881)	P value
Age (years)	58.5 ± 9.6	56.9 ± 10.5	< 0.001
Men	611 (82.8)	4,676 (79.5)	0.036
Year of diagnosis			< 0.001
2005–2007	135 (18.3)	1,625 (27.6)	
2008–2010	222 (30.1)	2,171 (36.9)	
2011–2013	381 (51.6)	2,085 (35.5)	
Etiology			0.46
Hepatitis B*	563 (76.3)	4,466 (75.9)	
Hepatitis C	78 (10.6)	562 (9.6)	
Others	97 (13.1)	853 (14.5)	
Child-Pugh class			< 0.001
A	672 (91.1)	4,930 (83.8)	
B	65 (8.8)	830 (14.1)	
C	1 (0.1)	121 (2.1)	
ALBI grade			
1	456 (61.8)	3,053 (51.9)	
2	269 (36.4)	2,518 (42.8)	
3	13 (1.8)	310 (5.3)	
BCLC stage			< 0.001
0	136 (18.4)	884 (15.0)	
A	387 (52.4)	2,629 (44.7)	
B	100 (13.6)	661 (11.2)	
C	113 (15.3)	1,530 (26.0)	
D	2 (0.3)	177 (3.0)	
AFP (ng/ml)	23 (7–182)	40 (7–502)	< 0.001
PIVKA-II (mAU/ml)	39 (19–358)	57 (21–500)	< 0.001
Initial treatment			< 0.001
Resection	200 (27.1)	1,673 (28.4)	
Ablation	150 (20.3)	1,129 (19.2)	
TACE	359 (48.6)	2,266 (38.5)	
LT	3 (0.4)	127 (2.2)	
Others	25 (3.4)	309 (5.3)	
Best supportive care	1 (0.1)	377 (6.4)	
LT during follow-up	41 (5.6)	328 (5.6)	0.98

Abbreviations: MDT, multidisciplinary tumor board; ALBI, albumin-bilirubin; BCLC, Barcelona Clinic Liver Cancer; AFP, alpha-fetoprotein; PIVKA-II, protein induced by vitamin K absence or antagonist-II; TACE, transarterial chemoembolization; LT, liver transplantation. Values are expressed as mean ± standard deviation, median (quartile) or number (%).

*Include 59 patients with hepatitis B virus/hepatitis C virus co-infection.

When compared, there was no significance difference in the proportion of patients receiving BCLC guideline recommended treatment between MDT and non-MDT group (54.7% vs. 53.7%, p = 0.58). However, when stratified according to tumor stage, less patients received BCLC guideline recommended treatment in BCLC stage 0 (69.1% vs. 83.1%, p = 0.001), BCLC stage A (58.1% vs. 65.4%, p = 0.005), and BCLC stage C (5.3% vs. 11.8%, p = 0.035) in MDT group, and more patients received BCLC guideline recommended treatment in BCLC stage B (78.0% vs. 69.0%) in MDT group.

**Fig 1:**
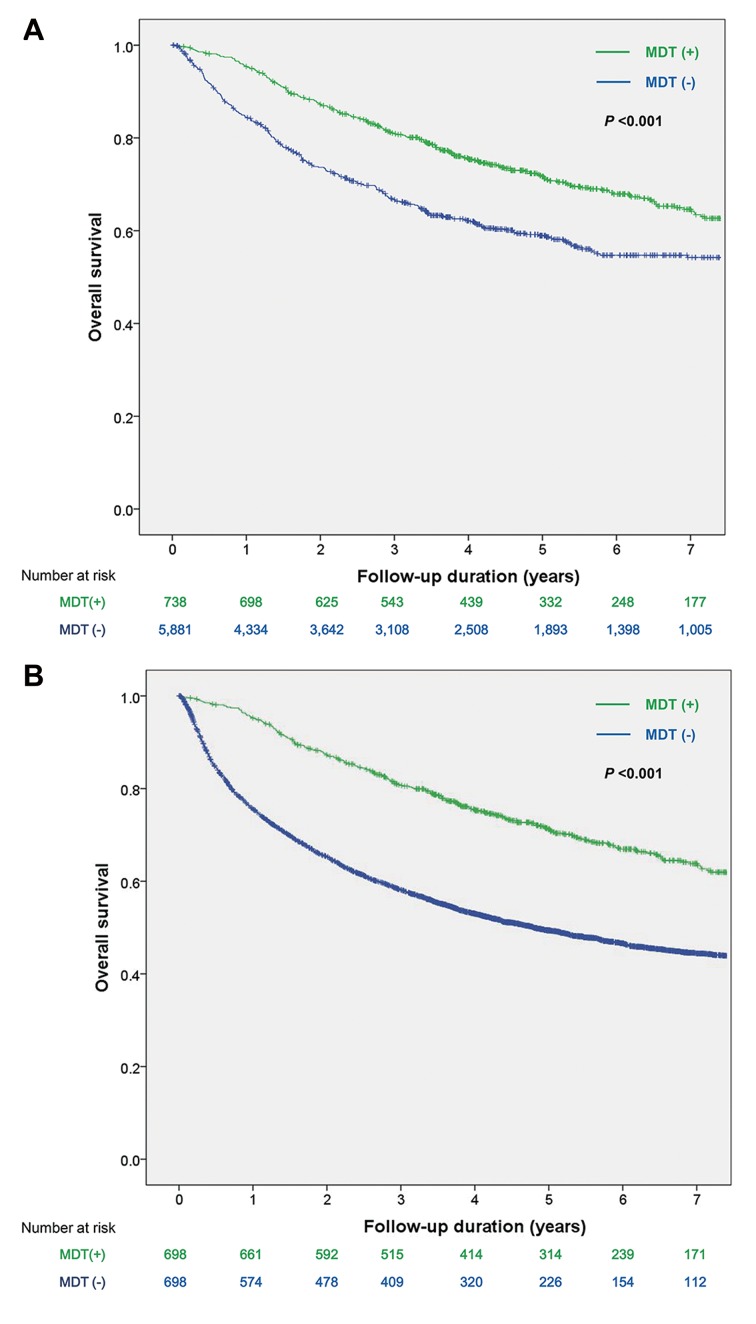
Kaplan-Meier survival curves of patients with hepatocellular carcinoma according to the management through multidisciplinary tumor board in the entire cohort (A) and in the exactly matched cohort (B). Abbreviations: MDT, multidisciplinary tumor board.

During the median follow-up of 3.5 years (range: 0.1–12.4 years), 3,266 patients (49.3%) died. The 1-, 3- and 5-year OS rates were 77.8%, 60.7% and 51.9%, respectively. The survival rate was significantly higher in patients who were managed via MDT compared with those who were not (71.2% vs. 49.4% at 5-year, P < 0.001, Fig 1A), with an adjusted HR of 0.47 (95% confidence interval [CI]; 0.41–0.53). MDT management was an independent factor for OS along with sex, etiology, ALBI grade, BCLC stage, AFP level, PIVKA-II level, and initial treatment modality (Table 2).

**Table 2:** Factors associated with overall survival.

	Un-adjusted		Multivariable-adjusted	
	HR (95% CI)	P value	HR (95% CI)	P value
MDT care (vs. no)	0.51 (0.45–0.58)	< 0.001	0.47 (0.41–0.53)	< 0.001
Age (/year)	1.00 (0.99–1.01)	0.61	0.99 (0.99–1.00)	0.73
Male (vs. female)	1.21 (1.10–1.32)	< 0.001	1.19 (1.08–1.31)	< 0.001
Etiology				
Hepatitis B	Reference		Reference	
Hepatitis C	1.14 (1.03–1.28)	0.015	1.14 (1.01–1.29)	0.029
Others	1.27 (1.15–1.39)	< 0.001	1.09 (0.99–1.21)	0.076
ALBI grade				
Grade 1	Reference		Reference	
Grade 2	1.93 (1.79–2.07)	< 0.001	1.67 (1.55–1.81)	< 0.001
Grade 3	2.39 (2.07–2.76)	< 0.001	1.91 (1.61–2.27)	< 0.001
BCLC stage				
0	Reference		Reference	
A	2.14 (1.84–2.48)	< 0.001	1.64 (1.40–1.92)	< 0.001
B	4.33 (3.67–5.11)	< 0.001	2.11 (1.76–2.53)	< 0.001
C	10.5 (9.05–12.1)	< 0.001	3.87 (3.26–4.60)	< 0.001
D	8.32 (6.70–10.3)	< 0.001	3.06 (2.36–3.95)	< 0.001
AFP (log_e_ ng/ml)	1.22 (1.21–1.23)	< 0.001	1.09 (1.08–1.11)	< 0.001
PIVKA-II (log_e_ mAU/ml)	1.40 (1.38–1.42)	< 0.001	1.17 (1.14–1.19)	< 0.001
Initial treatment				
Best supportive care	Reference		Reference	
Resection	0.03 (0.02–0.03)	< 0.001	0.07 (0.06–0.08)	< 0.001
Ablation	0.04 (0.03–0.04)	< 0.001	0.13 (0.10–0.15)	< 0.001
TACE	0.12 (0.11–0.14)	< 0.001	0.23 (0.20–0.26)	< 0.001
LT	0.02 (0.01–0.03)	< 0.001	0.03 (0.02–0.05)	< 0.001
Others	0.35 (0.30–0.41)	< 0.001	0.37 (0.31–0.44)	< 0.001

Abbreviations: HR, hazard ratio; MDT, multidisciplinary tumor board; ALBI, albumin-bilirubin; BCLC, Barcelona Clinic Liver Cancer; TACE, transarterial chemoembolization; LT, liver transplantation; AFP, alpha-fetoprotein; PIVKA-II, protein induced by vitamin K absence or antagonist-II.

The baseline characteristics of the exactly matched cohort are shown in S1 Table. After age (in four categories), sex (men vs. women), year of diagnosis (2005–2007, 2008–2010, and 2011–2013), etiology (hepatitis B virus vs. other), ALBI grade (grade 1, 2, and 3), BCLC stage (stage 0, A, B, C, and D), AFP levels (<200 and ≥ 200 mg/dl) and LT during follow-up were exactly matched, the initial treatment modality varied between the patients who were and were not managed through MDT (S2 Table). Proportions of the patients who underwent resection, ablation or LT as a first-line treatment were lower for those who were managed via MDT than those who did not (48.1% vs. 55.9%). None of the patients who were managed via MDT received the best supportive care (0% vs. 3.7%). In the matched cohort, more proportion of patients received BCLC guideline recommended treatment in MDT group (61.7% vs. 55.0%, p = 0.011). When stratified according to tumor stage, less patients received BCLC guideline recommended treatment in BCLC stage 0 (69.6% vs. 81.6%, p = 0.027), BCLC stage A (58.2% vs. 65.5%, p = 0.017). Although not statistically significant, more proportion of patients received BCLC guideline recommended treatment in BCLC stage B (80.5% vs. 71.3%, p = 0.156) in MDT group, and less proportion of patients received BCLC guideline recommended treatment in BCLC stage C (5.7% vs. 12.4%, p = 0.092). The OS rate was significantly higher in the patients who were managed via MDT in the exactly matched cohort (71.7% vs. 58.9% at 5-years, P < 0.001, Fig 1B), with an HR of 0.67 (95% CI, 0.56–0.80).

**Fig 2:**
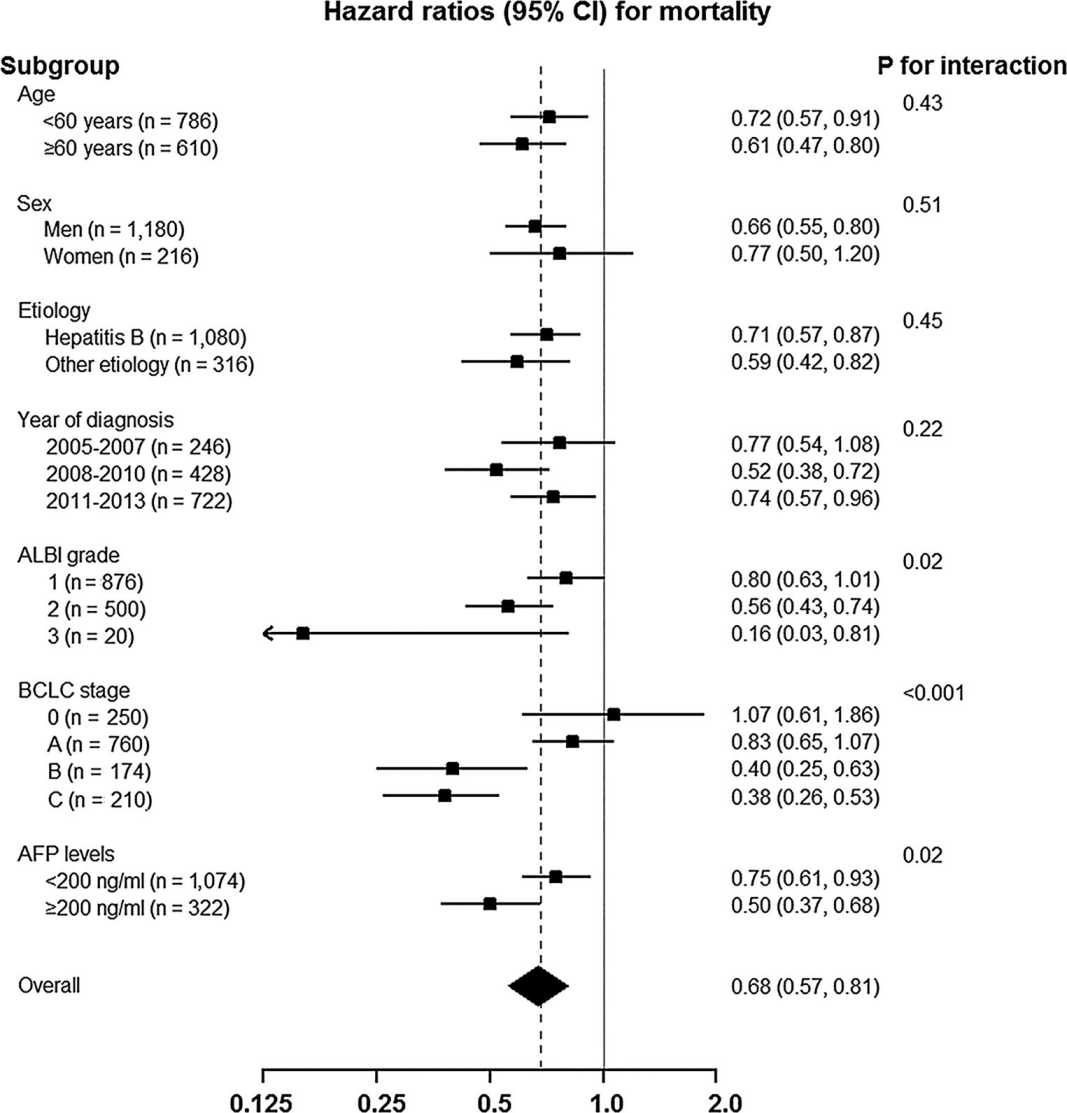
Hazard ratios for mortality comparing patients who were and were not managed through multidisciplinary tumor board in predefined subgroups at baseline. Abbreviations: ALBI, albumin-bilirubin; BCLC, Barcelona Clinic Liver Cancer; AFP, alpha-fetoprotein.

The association of the management via MDT and OS was consistent across most subgroups (Fig 2), and the association was stronger among patients with poor liver function (ALBI grade 2 or ALBI grade 3) at baseline (P for interaction = 0.02 for ALBI grade), intermediate or advanced BCLC stage (BCLC stage B or C) (P for interaction < 0.001 for BCLC stage), and higher serum AFP levels (≥200 ng/ml) (P for interaction = 0.02 for AFP levels). When stratified by major treatment modality (resection, ablation and transarterial chemoembolization), no difference in OS was observed between MDT and non-MDT group among patients who received resection in overall cohort (76.9% vs. 76.8%, p = 0.68) as well as in matched cohort (76.9% vs. 79.2%, p = 0.40). Among patients who received ablation, better OS was observed for patients who received MDT care in overall cohort (86.3% vs. 73.2%, p = 0.008), but not in matched cohort (86.6% vs. 73.9%, p = 0.21). Among patients who received transarterial chemoembolization, better OS was observed for patients who received MDT care in overall cohort (61.8% vs. 28.4%, p = 0.001) as well as in matched cohort (62.3% vs. 38.6%, p = 0.001) (S3 Table).

## Discussion

In this study, we observed better OS in HCC patients who had MDT management. Those received MDT management had different baseline characteristics than those who did not. Those received MDT management showed more preserved liver function, earlier tumor stage, lower levels of serum tumor markers, managed at more recent years, and had received more active treatment. However, MDT management was an independent factor associated with better OS in multivariable-adjusted model in the entire cohort. Also, MDT management was associated with better OS in the matched cohort, where the key factors related to patient outcome were exactly matched between patients who were and were not managed via MDT. The benefit of MDT management was observed across most pre-defined subgroups; especially, it was greater in those with poor liver function (ALBI grade 2 or ALBI grade 3), intermediate or advanced BCLC stage (BCLC stage B or C), or high AFP levels (≥200 ng/ml).

Our data is in line with previous studies which showed improved survival by MDT management [18–24]. The strength of this data is the relatively large sample size that enabled us to create exactly matched cohort. The better long-term outcome by MDT management in this study cannot be explained by differences in the baseline characteristics. It has been suggested that MDT approach is more beneficial when things are more complicated [13]. Interestingly, we observed that MDT management was more beneficial under more complex clinical situations (poorer liver function, intermediate or advanced tumor stage or higher AFP levels).

There are several plausible explanations underlying the improved survival associated with MDT management. It has been reported that MDT management can alters the direction of patient care by changes in imaging interpretation, revisions in diagnosis, and changes in treatment recommendations [23,27]. MDT management can also shorten the interval from diagnosis to treatment [22], and increase the odds of receiving treatment for HCC [18]. In a cohort study based on data from a MDT meeting dedicated to HCC, non-adherence to the MDT recommendation was one of the negative prognostic factors for OS [20]. MDT management has another advantage as patient is being managed by a group of specialists from multiple fields. HCC treatment selection is significantly influenced by provider subspeciality [28], that is, when assessed by only one specialist, optimal treatment selection for individual patient might be biased by provider’s experience and preferentiality [24]. A balanced treatment selection is ensured when specialists in each field openly discuss the risks and benefits of each treatment option. In this study, we could not present how many patients had change in imaging interpretation, revisions in diagnosis and changes in treatment recommendations, and how many patients had adherent to the MDT recommendation, as we have not recorded in this data prospectively. Notably, there was slight difference in treatment allocation between MDT and non-MDT group, careful interpretation is needed as this is an observational cohort study. Proportion receiving curative modalities (resection, ablation and LT) was similar between those in MDT and non-MDT group (49.2% vs. 50.3%). However, those receiving non-curative modalities (TACE and others) were much higher in MDT than non-MDT group (50.7% vs. 43.2%) in overall cohort. In matched cohort, less patients received curative modalities (49.4% vs. 56.4%) while more patients received non-curative modalities (50.6% vs. 39.8%) in MDT group. When further stratified according to BCLC stage, less proportion of patients in BCLC 0/A received BCLC-guideline recommended therapy (resection, ablation or LT) in MDT group, more proportion of patients in BCLC B received BCLC-guideline recommended therapy (TACE) in MDT group, and less proportion of patients in BCLC C received BCLC-guideline recommended therapy (sorafenib) in MDT group. However, in our practice, MDT usually discuss more complicated cases (e.g., small tumor located in segment 1, near major vessels or hilar area, which ablation or resection is not feasible or difficult), which explains why less proportion of patients received curative treatment in BCLC 0 or A in MDT group. Although MDT care may resulted in the revision in diagnosis, management or treatment which translated into better outcome, this study cannot answer whether observed better survival is from MDT care itself, or from selection of better patients with different characteristics in MDT group. Prospective evaluations are needed to see whether MDT care can really improve patient outcome, and to find out exact reasons behind the better outcome.

MDT management is not free of costs. It requires additional medical resources and costs. To date, MDT management is not reimbursed by National Health Insurance in Korea. MDT management has been practiced for free of costs in our institution till now. We had a limit for 15 cases per week for MDT discussion, which covers only about 10% of HCC patients. Expanding our MDT program was very challenging for multiple reasons including team capacities and cost issues. By simply gathering multiple academic disciplines together and allowing them to have a timely and open discussion, the outcome of HCC patients can be improved. In institutions where multiple academic disciplines are involved in HCC care, potential benefit of MDT care can be easily applied, as characteristics of MDT care do not require special equipment, rather, it requires only time and space for MDT member to have open discussion. A health care policy that supports MDT management is required to expand and sustain MDT management for HCC patients. Of note, we noticed that MDT management was especially helpful for those with poor liver function, intermediate or advanced tumor stage or high AFP levels. Given the restricted medical resources and cost, MDT management might be prioritized for a certain group of patients according to local regulations, team capacities and cost-benefit strategies.

This study has some other limitations. First, this is a retrospective cohort study. The decision to present a patient for discussion at MDT meeting was solely at the discretion of the provider in charge of the patient, and was not randomly selected patients. Hence, unidentified factors might be present that resulted in a better outcome for patients who received MDT care, including selection bias. Second, MDT management was not provided for every treatment among patients who have received multiple HCC treatment, but only a median of 1.4 times (min–max: 1–4) per each patient. HCC is notorious for its high recurrence rates. MDT management during all treatment courses of the patients might further benefit patients, which also requires prospective evaluation. Third, in subgroup analysis, those with ALBI grade 3 was small to have enough statistical power (n = 20). Fourth, the study was performed in South Korea, where hepatitis B is a major cause of HCC [29], and living-donor LT is a popular mode of LT [9]. Thus, generalizability of our findings to other regions with different etiologies and resources remains to be determined. Lastly, we observed better survival in MDT group for patients initially treated with transarterial chemoembolization, while this was not observed for patients initially treated with resection. Subsequent MDT management and treatment may explain this, as patients who are treated with transarterial chemoembolization usually experience higher recurrence than those treated with resection. However, exact reason for this is not clear.

In conclusion, management of HCC patients via MDT was associated with better survival. Especially, the benefit of MDT management was greater in those with poor liver function, intermediate or advanced tumor stage or higher AFP levels. MDT management might be prioritized for these groups of patients, if medical resources are of concern. MDT care has a potential to improve outcome of HCC patients where clinical situations are highly complex. Prospective evaluations are warranted and healthcare policy that can support MDT management for HCC patients are urgently required.

## Supporting information

S1 Table Baseline characteristics of the exactly matched cohort. (DOCX)

S2 Table Initial treatment of the exactly matched cohort. (DOCX)

S3 Table The association between multidisciplinary tumor board care and overall survival stratified by treatment modality. (DOCX)

## Abbreviations

HCC: Hepatocellular carcinoma
MDT: Multidisciplinary approach
OS: Overall survival
HR: Hazard ratio
CI: Confidence interval
BCLC: Barcelona Clinic Liver Cancer
AFP: alpha-fetoprotein
PIVKA-II: protein induced by vitamin K absence or antagonist-II
ALBI: albumin-bilirubin
ROC: Receiver operating curve

## References

[1] Bertuccio P, Turati F, Carioli G, Rodriguez T, La Vecchia C, Malvezzi M, et al Global trends and predictions in hepatocellular carcinoma mortality. J Hepatol. 2017; 67:302–9. 10.1016/j.jhep.2017.03.011 28336466

[2] Zhu RX, Seto WK, Lai CL, Yuen MF. Epidemiology of Hepatocellular Carcinoma in the Asia-Pacific Region. Gut Liver. 2016; 10:332–9. 10.5009/gnl15257 27114433 PMC4849684

[3] Kim BH, Park JW. Epidemiology of liver cancer in South Korea. Clin Mol Hepatol. 2018; 24:1–9. 10.3350/cmh.2017.0112 29249129 PMC5875192

[4] Blanc JF, Frulio N, Chiche L, Sempoux C, Annet L, Hubert C, et al Hepatocellular adenoma management: call for shared guidelines and multidisciplinary approach. Clin Res Hepatol Gastroenterol. 2015; 39:180–7. 10.1016/j.clinre.2014.10.003 25434466

[5] Lee HW, Sinn DH, Kang W, Gwak GY, Paik YH, Choi MS, et al Cause of Mortality for Hepatocellular Carcinoma Patients who were Diagnosed within the Milan Criteria. J Liver Cancer. 2016; 16:101–7. 10.17998/jlc.2016.16.2.101

[6] Heimbach JK, Kulik LM, Finn RS, Sirlin CB, Abecassis MM, Roberts LR, et al AASLD guidelines for the treatment of hepatocellular carcinoma. Hepatology. 2018; 67:358–80. 10.1002/hep.29086 28130846

[7] Korean Liver Cancer Study Group, National Cancer Center Korea. 2014 KLCSG-NCC Korea Practice Guideline for the Management of Hepatocellular Carcinoma. Gut Liver. 2015; 9:267–317. 10.5009/gnl14460 25918260 PMC4413964

[8] Moon H, Choi JE, Lee IJ, Kim TH, Kim SH, Ko YH, et al All-treatment array of hepatocellular carcinoma from initial diagnosis to death: observation of cumulative treatments. J Cancer Res Clin Oncol. 2017; 143:2327–39. 10.1007/s00432-017-2480-9 28744575 PMC5640756

[9] Lee HW, Suh KS. Liver transplantation for advanced hepatocellular carcinoma. Clin Mol Hepatol. 2016; 22:309–18. 10.3350/cmh.2016.0042 27729631 PMC5066382

[10] European Association for the Study of the Liver, European Organisation for Research and Treatment of Cancer. EASL-EORTC clinical practice guidelines: management of hepatocellular carcinoma. J Hepatol. 2012; 56:908–43. 10.1016/j.jhep.2011.12.001 22424438

[11] Yu SJ. A concise review of updated guidelines regarding the management of hepatocellular carcinoma around the world: 2010–2016. Clin Mol Hepatol. 2016; 22:7–17. 10.3350/cmh.2016.22.1.7 27044761 PMC4825164

[12] Kim KM, Sinn DH, Jung SH, Gwak GY, Paik YH, Choi MS, et al The recommended treatment algorithms of the BCLC and HKLC staging systems: does following these always improve survival rates for HCC patients? Liver Int. 2016; 36:1490–7. 10.1111/liv.13107 26936471

[13] Plsek PE, Greenhalgh T. Complexity science: The challenge of complexity in health care. BMJ. 2001; 323:625–8. 11557716 10.1136/bmj.323.7313.625 PMC1121189

[14] European Partnership Action Against Cancer Consensus Group, Borras JM, Albreht T, Audisio R, Briers E, Casali P, et al Policy statement on multidisciplinary cancer care. Eur J Cancer. 2014; 50:475–80. 10.1016/j.ejca.2013.11.012 24321260

[15] Plsek PE, Wilson T. Complexity, leadership, and management in healthcare organisations. BMJ. 2001; 323:746–9. 11576986 10.1136/bmj.323.7315.746 PMC1121291

[16] Italian Association for the Study of the Liver, Aisf Expert Panel, Aisf Coordinating Committee, Bolondi L, Cillo U, Colombo M, et al Position paper of the Italian Association for the Study of the Liver (AISF): the multidisciplinary clinical approach to hepatocellular carcinoma. Dig Liver Dis. 2013; 45:712–23. 10.1016/j.dld.2013.01.012 23769756

[17] De Ieso PB, Coward JI, Letsa I, Schick U, Nandhabalan M, Frentzas S, et al A study of the decision outcomes and financial costs of multidisciplinary team meetings (MDMs) in oncology. Br J Cancer. 2013; 109:2295–300. 10.1038/bjc.2013.586 24084764 PMC3817328

[18] Agarwal PD, Phillips P, Hillman L, Lucey MR, Lee F, Mezrich JD, et al Multidisciplinary Management of Hepatocellular Carcinoma Improves Access to Therapy and Patient Survival. J Clin Gastroenterol. 2017; 51:845–9. 10.1097/mcg.0000000000000825 28877082

[19] Chang TT, Sawhney R, Monto A, Davoren JB, Kirkland JG, Stewart L, et al Implementation of a multidisciplinary treatment team for hepatocellular cancer at a Veterans Affairs Medical Center improves survival. HPB (Oxford). 2008; 10:405–11. 10.1080/13651820802356572 19088925 PMC2597312

[20] Charriere B, Muscari F, Maulat C, Bournet B, Bonnet D, Bureau C, et al Outcomes of patients with hepatocellular carcinoma are determined in multidisciplinary team meetings. J Surg Oncol. 2017; 115:330–6. 10.1002/jso.24500 27813094

[21] Zaky S, Makhlouf NA, Abdel Malek MO, Bakheet AA, Seif HM, Hamza HM, et al Multidisciplinary decision making in the management of hepatocellular carcinoma: A hospital-based study. Turk J Gastroenterol. 2015; 26:498–505. 10.5152/tjg.2015.0158 26510081

[22] Yopp AC, Mansour JC, Beg MS, Arenas J, Trimmer C, Reddick M, et al Establishment of a multidisciplinary hepatocellular carcinoma clinic is associated with improved clinical outcome. Ann Surg Oncol. 2014; 21:1287–95. 10.1245/s10434-013-3413-8 24318095 PMC5612826

[23] Zhang J, Mavros MN, Cosgrove D, Hirose K, Herman JM, Smallwood-Massey S, et al Impact of a single-day multidisciplinary clinic on the management of patients with liver tumours. Curr Oncol. 2013; 20:e123–31. 10.3747/co.20.1297 23559879 PMC3615863

[24] Gashin L, Tapper E, Babalola A, Lai KC, Miksad R, Malik R, et al Determinants and outcomes of adherence to recommendations from a multidisciplinary tumour conference for hepatocellular carcinoma. HPB (Oxford). 2014; 16:1009–15. 10.1111/hpb.12280 24888730 PMC4487752

[25] Serper M, Taddei TH, Mehta R, D'Addeo K, Dai F, Aytaman A, et al Association of Provider Specialty and Multidisciplinary Care With Hepatocellular Carcinoma Treatment and Mortality. Gastroenterology. 2017; 152:1954–64. 10.1053/j.gastro.2017.02.040 28283421 PMC5664153

[26] Johnson PJ, Berhane S, Kagebayashi C, Satomura S, Teng M, Reeves HL, et al Assessment of liver function in patients with hepatocellular carcinoma: a new evidence-based approach-the ALBI grade. J Clin Oncol. 2015; 33:550–8. 10.1200/jco.2014.57.9151 25512453 PMC4322258

[27] Soares KC, Cosgrove DC, Herman JM, Pawlik TM. Multidisciplinary clinic in the management of hepatocellular carcinoma. Ann Surg Oncol. 2014; 21:1059–61. 10.1245/s10434-013-3419-2 24318097 PMC4005362

[28] Nathan H, Segev DL, Bridges JF, Massie AB, Cameron AM, Hirose K, et al Influence of nonclinical factors on choice of therapy for early hepatocellular carcinoma. Ann Surg Oncol. 2013; 20:448–56. 10.1245/s10434-012-2619-5 22941170

[29] Cho EJ, Kim SE, Suk KT, An J, Jeong SW, Chung WJ, et al Current status and strategies for hepatitis B control in Korea. Clin Mol Hepatol. 2017; 23:205–11. 10.3350/cmh.2017.0104 28942624 PMC5628005

