# Supplementary material for: Multidisciplinary approach is associated with improved survival of hepatocellular carcinoma patients
Source: PLoS One. 2019 Jan 14;14(1):e0210730. doi: 10.1371/journal.pone.0210730 (PMC6331107; doi:10.1371/journal.pone.0210730)
Supplement: S1 Table — (DOCX) [file pone.0210730.s001.docx]

**S1 Table. Baseline characteristics of the exactly matched cohort**

|  | | **With MDT care**  **(n = 698)** | | **Without MDT care**  **(n = 698)** | **P value** |
| --- | --- | --- | --- | --- | --- |
| Age (years) | |  | |  | 1.00 |
| <50 | | 111 (15.9) | | 111 (15.9) |  |
| 50-59 | | 282 (40.4) | | 282 (40.4) |  |
| 60-69 | | 212 (30.4) | | 212 (30.4) |  |
| ≥70 | | 93 (13.3) | | 93 (13.3) |  |
| Male | | 590 (84.5) | | 590 (84.5) | 1.00 |
| Year of diagnosis | |  | |  | 1.00 |
| 2005-2007 | | 123 (17.6) | | 123 (17.6) |  |
| 2008-2010 | | 214 (30.7) | | 214 (30.7) |  |
| 2011-2013 | | 361 (51.7) | | 361 (51.7) |  |
| Hepatitis B | | 540 (77.4) | | 540 (77.4) | 1.00 |
| ALBI grade | |  | |  | 1.00 |
| 1 | | 438 (62.8) | | 438 (62.8) |  |
| 2 | | 250 (35.8) | | 250 (35.8) |  |
| 3 | | 10 (1.4) | | 10 (1.4) |  |
| BCLC stage | |  | |  | 1.00 |
| 0 | | 125 (17.9) | | 125 (17.9) |  |
| A | | 380 (54.4) | | 380 (54.4) |  |
| B | | 87 (12.5) | | 87 (12.5) |  |
| C | | 105 (15.0) | | 105 (15.0) |  |
| D | | 1 (0.1) | | 1 (0.1) |  |
| AFP levels (ng/mL) | |  | |  | 1.00 |
| <200 | | 537 (76.9) | | 537 (76.9) |  |
| ≥200 | | 161 (23.1) | | 161 (23.1) |  |
| LT during follow-up | 28 (4.0) | 28 (4.0) | | 1.00 | |

Abbreviations: MDT, multidisciplinary tumor board; ALBI, albumin-bilirubin; BCLC, Barcelona Clinic Liver Cancer; AFP, alpha-fetoprotein; LT, liver transplantation**.**
