# Supplementary material for: Multidisciplinary approach is associated with improved survival of hepatocellular carcinoma patients
Source: PLoS One. 2019 Jan 14;14(1):e0210730. doi: 10.1371/journal.pone.0210730 (PMC6331107; doi:10.1371/journal.pone.0210730)
Supplement: S2 Table — (DOCX) [file pone.0210730.s002.docx]

**S2 Table. Initial treatment of the exactly matched cohort**

|  | **With MDT care**  **(n = 698)** | | **Without MDT care**  **(n = 698)** | **P value** |
| --- | --- | --- | --- | --- |
| Initial treatment |  | |  | < 0.001 |
| Resection | 190 (27.2) | | 228 (32.7) |  |
| Ablation | 144 (20.6) | | 151 (21.6) |  |
| TACE | 338 (48.4) | | 260 (37.2) |  |
| LT | 2 (0.3) | | 11 (1.6) |  |
| Others | 24 (3.4) | | 22 (3.2) |  |
| Best supportive care | 0 | 26 (3.7%) | |  |

Abbreviations: MDT, multidisciplinary tumor board; TACE, transarterial chemoembolization; LT, liver transplantation**.**
