# Supplementary material for: Multidisciplinary approach is associated with improved survival of hepatocellular carcinoma patients
Source: PLoS One. 2019 Jan 14;14(1):e0210730. doi: 10.1371/journal.pone.0210730 (PMC6331107; doi:10.1371/journal.pone.0210730)
Supplement: S3 Table — (DOCX) [file pone.0210730.s003.docx]

**S3 Table. The association between multidisciplinary tumor board care and overall survival stratified by treatment modality**

|  | MDT | Non-MDT | P value | Adjusted HR  (95% CI) | P value |
| --- | --- | --- | --- | --- | --- |
| Overall cohort |  |  |  |  |  |
| Resection | 76.9% | 76.8% | 0.68 | 1.12 (0.84-1.49) | 0.41 |
| Ablation | 86.3% | 73.2% | 0.008 | 0.59 (0.41-0.84) | 0.04 |
| TACE | 61.8% | 28.4% | 0.001 | 0.42 (0.36-0.50) | 0.001 |
| Matched cohort |  |  |  |  |  |
| Resection | 76.9% | 79.2% | 0.40 | 1.23 (0.81-1.85) | 0.31 |
| Ablation | 86.6% | 73.9% | 0.21 | 0.72 (0.44-1.17) | 0.19 |
| TACE | 62.3% | 38.6% | 0.001 | 0.44 (0.34-0.55) | 0.001 |

Abbreviations: MDT, multidisciplinary tumor board; TACE, transarterial chemoembolization; HR, hazard ratio; CI, confidence interval. Adjusted for age, sex , etiology, albumin-bilirubin grade, Barcelona Clinic Liver Cancer stage and serum alphafetoprotein and PIVKA-II levels.
